# Extent of resection and survival in IDH-wildtype glioblastoma: interaction with MGMT status and chemoradiation

**DOI:** 10.1007/s11060-026-05725-x

**Published:** 2026-07-23

**Authors:** Noah B. Drewes, Lara Koutah, Rishi Jain, Jack Carnduff, Kayla Chin, Kristin R. Delfino, Jeffrey W. Cozzens, Bruce M. Frankel

**Affiliations:** 1 Division of Neurosurgery, Department of Surgery, Southern Illinois University School of Medicine, 801 N. Rutledge St., PO Box 19620, Springfield, IL 62702 USA; 2 Department of Neurological Surgery, Feinberg School of Medicine, Northwestern University, Chicago, IL 60611 USA; 3 Center for Clinical Research, Southern Illinois University School of Medicine, Springfield, IL USA

**Keywords:** Glioblastoma, IDH-wildtype, Extent of resection, MGMT promoter methylation, Chemoradiation, Overall survival

## Abstract

**Purpose:**

Whether gross total resection (GTR) remains associated with survival among MGMT-methylated IDH-wildtype glioblastoma patients receiving chemoradiation remains uncertain. We evaluated whether GTR versus less-than-GTR (< GTR) was associated with overall survival across MGMT promoter methylation and chemoradiation strata.

**Methods:**

We retrospectively reviewed 261 patients undergoing biopsy or resection for newly diagnosed IDH-wildtype glioblastoma at a single institution from 2010 to 2024. Extent of resection was dichotomized as GTR versus < GTR, and chemoradiation was coded as receipt of postoperative radiation and temozolomide. Overall survival was assessed using Kaplan-Meier analysis and multivariable Cox models with prespecified EOR × MGMT × chemoradiation interaction testing, adjusted for age, KPS, tumor location, and mFI-5.

**Results:**

GTR was associated with longer overall survival than < GTR in the overall cohort and within all four MGMT × chemoradiation strata. MGMT-methylated patients receiving chemoradiation had a median overall survival of 17.02 months after GTR versus 10.65 months after < GTR (log-rank *p* = 0.001), and the model-derived adjusted hazard ratio for < GTR versus GTR was 2.12 (95% CI 1.27–3.54; *p* = 0.004). The three-way EOR × MGMT × chemoradiation interaction was not significant (likelihood-ratio *p* = 0.309). In a 6-week sensitivity analysis, all survival associations remained, but EOR × chemoradiation was no longer significant.

**Conclusion:**

GTR was associated with longer survival across all MGMT and chemoradiation-defined subgroups, including MGMT-methylated patients receiving chemoradiation. These findings are consistent with maximal safe resection when feasible but should be interpreted cautiously because of small subgroups as well as treatment-selection and immortal-time biases.

**Supplementary Information:**

The online version contains supplementary material available at https://doi.org/10.1007/s11060-026-05725-x.

## Introduction

Glioblastoma, the most common malignant primary brain tumor in adults, maintains a poor prognosis regardless of aggressive treatment [1]. Under the 2021 World Health Organization classification, glioblastoma is defined as an IDH-wildtype diffuse glioma, rendering molecularly defined studies increasingly important [2]. Established factors associated with survival are maximal safe resection, radiotherapy with temozolomide, and MGMT promoter methylation status [3–7].

Prior literature supports the association of extent of resection (EOR) with survival and progression, but definitions have been heterogeneous across studies, and many reports predated routine molecular classification [8–13]. Additionally, recent work has emphasized postoperative residual tumor, including both contrast-enhancing and non-contrast-enhancing tumor, as clinically relevant measures of resection beyond classic EOR definitions [4, 14, 15]. These data support continued interest in the contexts in which residual enhancing tumor remains prognostic.

The importance of gross total resection (GTR) may be questioned when the potential survival benefit of a greater resection is weighed against the risk of postoperative deficits, especially in a disease with an already diminished prognosis [16]. This uncertainty may be greatest in MGMT-methylated glioblastoma treated with chemoradiation, as favorable treatment biology may attenuate the survival difference associated with resection extent; however, residual enhancing tumor burden may remain prognostically important [17].

As such, whether the survival association of GTR persists across combined MGMT promoter methylation and chemoradiation strata remains uncertain [18–20]. Though large registry studies provide substantially greater power and generalizability, registry-derived EOR and treatment variables may be subject to heterogeneous definitions and misclassification and may lack preoperative functional data and detailed imaging-based tumor locations. Single-center cohorts can define these variables more directly, although with a smaller sample size. Therefore, we evaluated if the association of GTR versus less-than-GTR (< GTR) with overall survival differed by MGMT status and chemoradiation in an institutional IDH-wildtype glioblastoma cohort.

## Methods

### Study design and data source

A single-center, retrospective study was conducted on patients who underwent biopsy or resection of histopathologically confirmed IDH-wildtype glioblastoma between January 2010 and December 2024. Data concerning clinical, operative, radiographic, molecular, treatment, and survival variables were abstracted from a prospectively maintained institutional registry. This study was approved by the Institutional Review Board and Ethics Committee (IRB) at Southern Illinois University School of Medicine (approval number: 25–797).

### Patient selection

Patients who underwent surgical management for newly diagnosed IDH-wildtype glioblastoma were eligible for inclusion. Cases were not included when the initial tumor surgery had been performed at another center, there was a lack of evaluable MRI, the operation took place before 2010, diagnosis occurred more than 3 months after symptom onset, or the tumor had features outside the intended study population, including infratentorial or spinal location, diffuse/non-enhancing disease only, multifocal or multicentric disease, or malignant transformation from a previously diagnosed lower-grade glioma.

### Clinical and demographic variables

Age was recorded at the time of first histopathologic diagnosis. Baseline functional status was evaluated using Karnofsky Performance Status (KPS) and Eastern Cooperative Oncology Group (ECOG) and was abstracted from chart review. Because KPS and ECOG measure overlapping aspects of functional status, KPS was selected for the primary multivariable models. KPS was dichotomized as ≥ 90 versus < 90.

Frailty and comorbidity burden were assessed using the preoperative 5-factor modified frailty index (mFI-5), which has been evaluated as a frailty measure in brain tumor cohorts, and was abstracted from chart review [21]. Patients were classified as having elevated frailty if mFI-5 was ≥ 2.

Tumor location was abstracted from preoperative imaging and operative reports. For the primary models, location was dichotomized as deep versus lobar. Deep location included tumors involving the thalamus/basal ganglia or corpus callosum. All other supratentorial tumor locations were categorized as lobar.

### Molecular variables

IDH1/IDH2 mutation status and MGMT promoter methylation status were obtained from clinical pathology reports when available. For tissue without routine molecular testing, archived tissue was retrospectively analyzed for IDH mutation and MGMT promoter methylation status. Only IDH-wildtype tumors were included in the final cohort. MGMT promoter status was categorized as methylated versus unmethylated/not methylated.

### Extent of resection

Extent of resection was determined using operative documentation and postoperative MRI. Patients undergoing resection underwent MRI brain with and without contrast within 48 h of surgery using thin-slice imaging. EOR was defined as the percentage of contrast-enhancing tumor removed. Cases with no residual postoperative contrast enhancement were classified as GTR. For cases with residual enhancement, preoperative contrast-enhancing tumor volume was compared with postoperative residual enhancing volume using Medtronic StealthStation software, and the percentage of tumor removed was recorded. EOR classifications were confirmed by a neuroradiologist and a second independent observer.

GTR was defined as removal of 100% of contrast-enhancing tumor. Near-total resection (NTR) was defined as < 100% but > 90% removal, subtotal resection (STR) as < 90% but > 50% removal, and minimal resection (MR) as < 50% removal or biopsy alone. For the primary analysis, EOR was dichotomized as GTR versus < GTR, with < GTR including near-total, subtotal, minimal resection, and biopsy. A sensitivity analysis was conducted to further stratify < GTR into NTR/STR (> 50% resection) and MR/biopsy (< 50% resection or biopsy).

### Treatment variables

Postoperative radiation therapy and chemotherapy receipt were abstracted from the medical record. At our institution, the intended postoperative treatment for newly diagnosed glioblastoma was fractionated external-beam radiotherapy to 60 Gy in 30 fractions over 6 weeks for patients younger than 70 years and hypofractionated radiotherapy to 40 Gy in 15 fractions over 3 weeks for patients aged 70 years or older. For either radiotherapy schedule, the intended concurrent chemotherapy was temozolomide 75 mg/m² orally daily, followed by adjuvant temozolomide for up to 6 monthly cycles when clinically appropriate.

For the primary analysis, chemoradiation (ChemoRT) was coded as “yes” only if the patient received both postoperative radiation therapy and temozolomide chemotherapy. Initiation was defined as the receipt of at least two radiotherapy fractions and at least two doses of concurrent temozolomide. Patients who received only one modality or neither were classified as no ChemoRT. This variable captured postoperative treatment received rather than randomized treatment assignment or baseline treatment intent. Classification as ChemoRT did not require completion of the full intended treatment course.

### Outcome

The primary outcome was overall survival (OS), defined as the time from first histopathologic diagnosis to death or last known follow-up. Death dates were obtained from the medical record or public records.

### Statistical analysis

Baseline characteristics were summarized using medians with interquartile ranges or counts with percentages. Age was compared using Wilcoxon rank-sum tests for two-group comparisons and Kruskal-Wallis tests were used for four-group comparisons. Chi-square or Fisher exact tests were used for categorical variables.

Survival was estimated using Kaplan-Meier methods, with GTR and < GTR compared within each MGMT × chemoradiation stratum using log-rank tests. Cox proportional hazards models evaluated whether the association between EOR and survival differed by MGMT status and chemoradiation. The full model included EOR, MGMT, chemoradiation, all two-way interactions, the EOR × MGMT × chemoradiation interaction, and prespecified covariates including age per 10 years, KPS ≥ 90, deep/corpus callosum location, and mFI-5 ≥ 2. The three-way interaction was evaluated using a Wald test and nested likelihood-ratio test. Because it was not supported, the reduced model retaining main effects and all two-way interactions plus covariates was used. Two-way interactions were evaluated using nested likelihood-ratio tests. Stratum-specific adjusted HRs for < GTR versus GTR were derived from the reduced model.

Proportional hazards were assessed using the Grambsch-Therneau test on scaled Schoenfeld residuals with log-transformed time. Because ChemoRT was coded as postoperative treatment received, a sensitivity analysis excluded patients who died within 6 weeks after diagnosis to reduce bias from early mortality and immortal time. An additional sensitivity analysis recategorized EOR as GTR, NTR/STR, or MR/biopsy. Kaplan-Meier curves and three-group log-rank tests were generated for overall and MGMT × ChemoRT subgroups. Adjusted Cox models used the same covariates for NTR/STR and MR/biopsy versus GTR. A separate analysis compared only GTR and NTR/STR to ensure the < GTR association persisted after excluding MR/biopsy cases. Cox ties used the Efron method; tests were two-sided with *p* < 0.05 considered significant. Analyses were performed in R version 4.5.3.

## Results

**Fig. 1:**
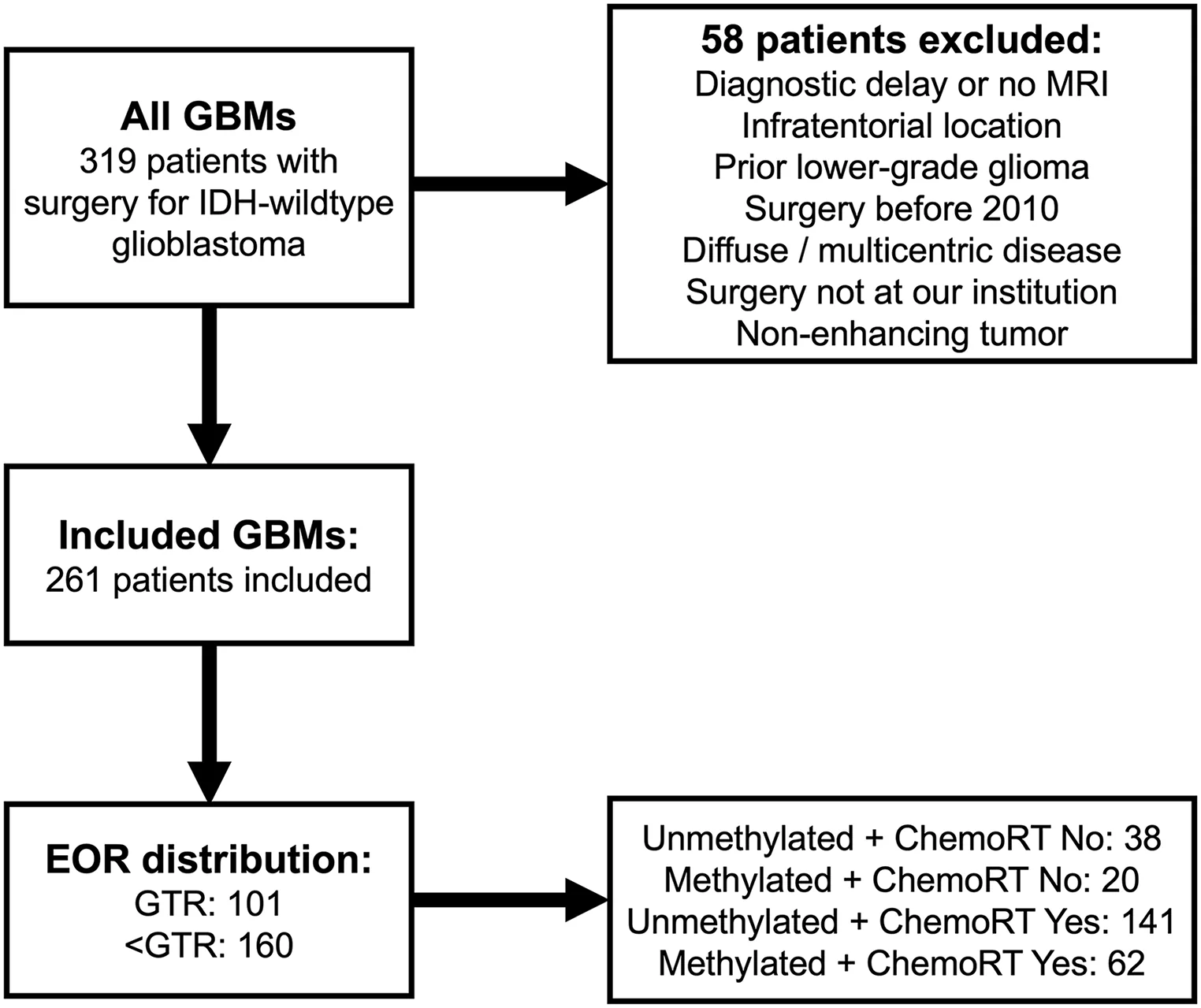
Cohort overview for the final analytic cohort of patients with newly diagnosed IDH-wildtype glioblastoma. Patients were categorized by extent of resection and stratified by MGMT promoter methylation status and receipt of chemoradiation. Abbreviations: *ChemoRT*, chemoradiation; *EOR*, extent of resection; *GBM*, glioblastoma; *GTR*, gross total resection; *IDH*, isocitrate dehydrogenase; *MGMT*, O^6^-methylguanine-DNA methyltransferase; *< GTR*, less-than-gross-total resection

A total of 261 patients with IDH-wildtype glioblastoma were included in the final analytic cohort, and a representative flow diagram is demonstrated in Fig. 1. Baseline characteristics were compared between patients undergoing GTR and those undergoing < GTR. Among the overall cohort, patients undergoing GTR were younger (*p* = 0.036), had better preoperative performance status (*p* < 0.001), were less likely to have deep/corpus callosum tumors (*p* < 0.001), and were more likely to receive chemoradiation (ChemoRT) (*p* < 0.001) than patients undergoing < GTR, while MGMT methylation status was similar between groups (*p* = 0.728). These findings are shown in Table 1. Baseline characteristics by MGMT × ChemoRT subgroup are summarized in Supplementary Table 1.

**Table 1:** Baseline characteristics by extent of resection

Characteristic	GTR (*n* = 101)	< GTR (*n* = 160)	*P*-Value
Age, years, median [IQR]	65.5 [57.1–71.5]	67.4 [61.5–75.2]	0.036^*^
KPS ≥ 90, n (%)	95 (94.1)	119 (74.4)	< 0.001^*^
mFI-5 ≥ 2, n (%)	20 (19.8)	48 (30.0)	0.068
Deep or CC location, n (%)	0 (0.0)	42 (26.3)	< 0.001^*^
MGMT methylated, n (%)	33 (32.7)	49 (30.6)	0.728
Received chemoradiation, n (%)	91 (90.1)	112 (70.0)	< 0.001^*^

Abbreviations: *CC*, corpus callosum; *EOR*, extent of resection; *GTR*, gross total resection; *IQR*, interquartile range; *KPS*, Karnofsky Performance Status; *mFI-5*, 5-factor modified frailty index; *MGMT*, O^6^-methylguanine-DNA methyltransferase; *< GTR*, less-than-gross-total resection

* denotes *p* < 0.05

**Fig. 2:**
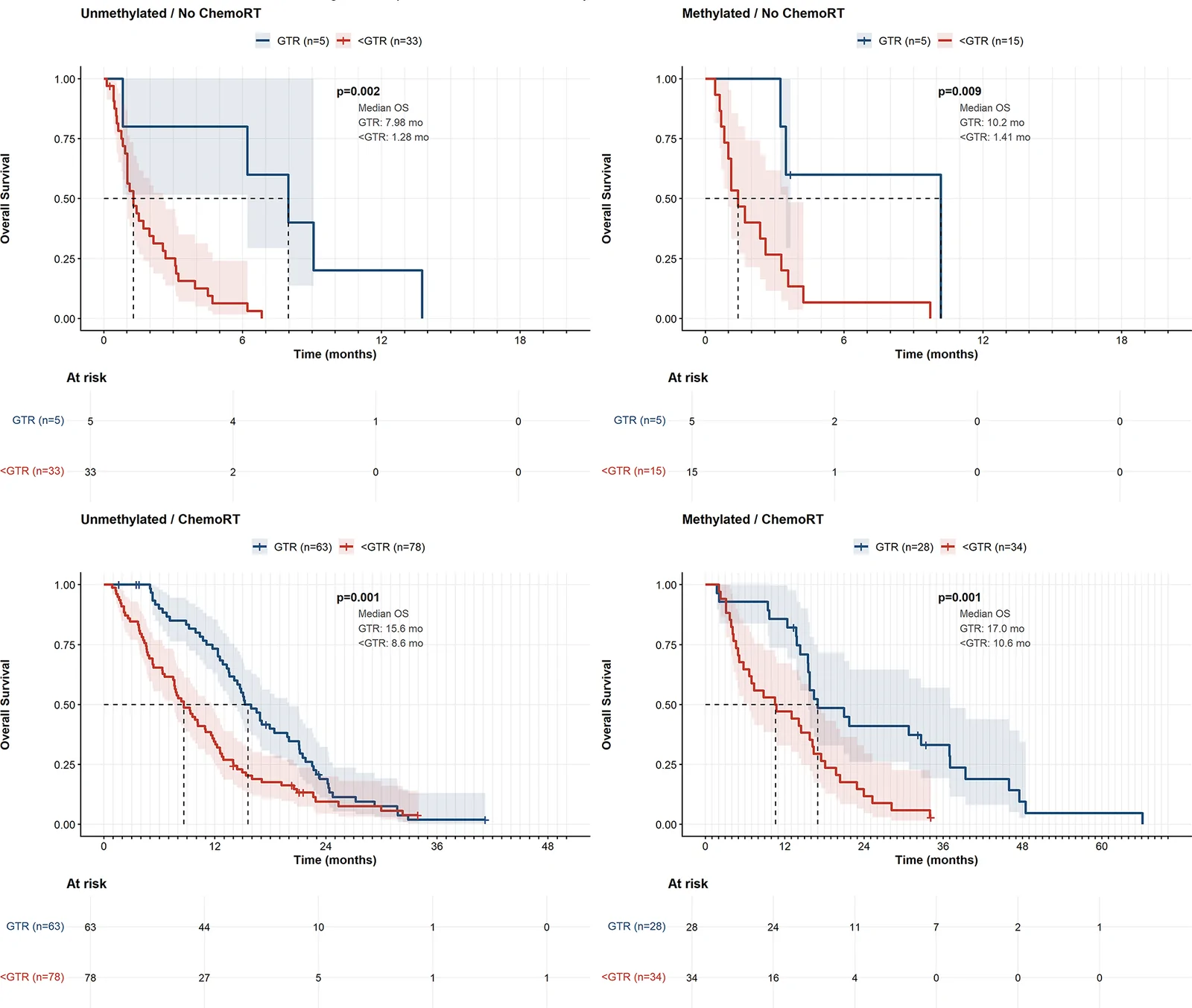
Kaplan-Meier overall survival curves comparing GTR and < GTR within each MGMT promoter methylation and chemoradiation stratum. P values are from log-rank tests, shaded bands represent 95% confidence intervals, and risk tables show the number at risk by extent-of-resection group. Abbreviations: *ChemoRT*, chemoradiation; *CI*, confidence interval; *EOR*, extent of resection; *GTR*, gross total resection; *MGMT*, O^6^-methylguanine-DNA methyltransferase; *OS*, overall survival; *< GTR*, less-than-gross-total resection

In a Kaplan-Meier analysis, GTR was associated with longer overall survival than < GTR, with median OS of 15.6 months after GTR versus 4.8 months after < GTR (log-rank *p* < 0.0001), which is demonstrated in Supplementary Fig. 1. This association was observed within all four MGMT × ChemoRT subgroups (Fig. 2; Supplementary Table 2). In the methylated/ChemoRT subgroup, median OS was 17.02 months after GTR versus 10.65 months after < GTR (*p* = 0.001). Although GTR was favored in all strata, curve separation appeared smaller among ChemoRT-treated patients. Stratified Kaplan-Meier curves and corresponding median OS estimates are shown in Fig. 2 and Supplementary Table 2.

To evaluate whether the association between EOR and survival differed by MGMT status and ChemoRT, we first fit a full Cox model including EOR, MGMT, ChemoRT, all two-way interactions, the EOR × MGMT × ChemoRT three-way interaction, and prespecified covariates of age per 10-year increase, KPS ≥ 90 versus < 90, deep/corpus callosum versus other tumor location, and mFI-5 ≥ 2 versus < 2. The three-way interaction was not significant (Wald *p* = 0.304; likelihood-ratio *p* = 0.309), and therefore the reduced two-way interaction model was used for primary inference. In likelihood-ratio testing, the EOR × ChemoRT interaction was significant (*p* = 0.033; corresponding reduced-model coefficient *p* = 0.045), whereas the EOR × MGMT interaction (*p* = 0.334), MGMT × ChemoRT interaction (*p* = 0.896), and all-two-way interaction block (*p* = 0.148) were not significant. These findings suggest possible attenuation of the EOR-survival association among patients receiving ChemoRT. Interaction testing is summarized in Table 2, Panel A.

In the reduced multivariable Cox model, < GTR was associated with worse survival in the reference group, which consisted of unmethylated/no-ChemoRT (HR 3.73, 95% CI 1.68–8.30, *p* = 0.001). ChemoRT receipt, KPS ≥ 90, older age, and deep/corpus callosum location were also significantly associated with survival, whereas MGMT and mFI-5 ≥ 2 were not significant in this interaction model (Table 2, Panel B).

**Table 2:** Primary Cox model and interaction testing

Model Comparison			LRT χ ²		df	P-Value
**Panel A. Interaction Testing Strategy**						
Three-way interaction						
Full (3-way) vs reduced (2-way)			1.04		1	0.309
Two-way interactions (each vs main effects only)						
EOR × MGMT			0.93		1	0.334
EOR × Chemoradiation			4.55		1	0.033^*^
MGMT × Chemoradiation			0.02		1	0.896
All two-way interactions (block)			5.35		3	0.148

**Parameter**	**β**	**SE**	**HR**	**95% CI lower**	**95% CI upper**	**P-Value**
**Panel B. Reduced Two-Way Interaction Cox Model**						
Primary predictors						
EOR: <GTR vs GTR	1.317	0.408	3.73	1.68	8.3	0.001^*^
MGMT methylated vs unmethylated	-0.599	0.391	0.55	0.26	1.18	0.126
Chemoradiation: yes vs no	-1.269	0.388	0.28	0.13	0.6	0.001^*^
Interaction terms						
EOR × MGMT methylated	0.272	0.305	1.31	0.72	2.39	0.373
EOR × Chemoradiation	-0.837	0.419	0.43	0.19	0.98	0.045^*^
MGMT methylated × Chemoradiation	0.047	0.346	1.05	0.53	2.07	0.892
Covariates						
Age, per 10-year increment	0.179	0.063	1.2	1.06	1.35	0.005^*^
KPS ≥ 90 vs < 90	-0.504	0.189	0.6	0.42	0.87	0.008^*^
Deep or CC location vs other	0.69	0.203	1.99	1.34	2.97	0.001^*^
mFI-5 ≥ 2 vs < 2	0.126	0.163	1.13	0.82	1.56	0.44

Abbreviations: β, regression coefficient; CC, corpus callosum; CI, confidence interval; EOR, extent of resection; GTR, gross total resection; HR, hazard ratio; KPS, Karnofsky Performance Status; LRT, likelihood-ratio test; mFI-5, 5-factor modified frailty index; MGMT, O^6^-methylguanine-DNA methyltransferase; SE, standard error

* denotes p < 0.05

**Fig. 3:**
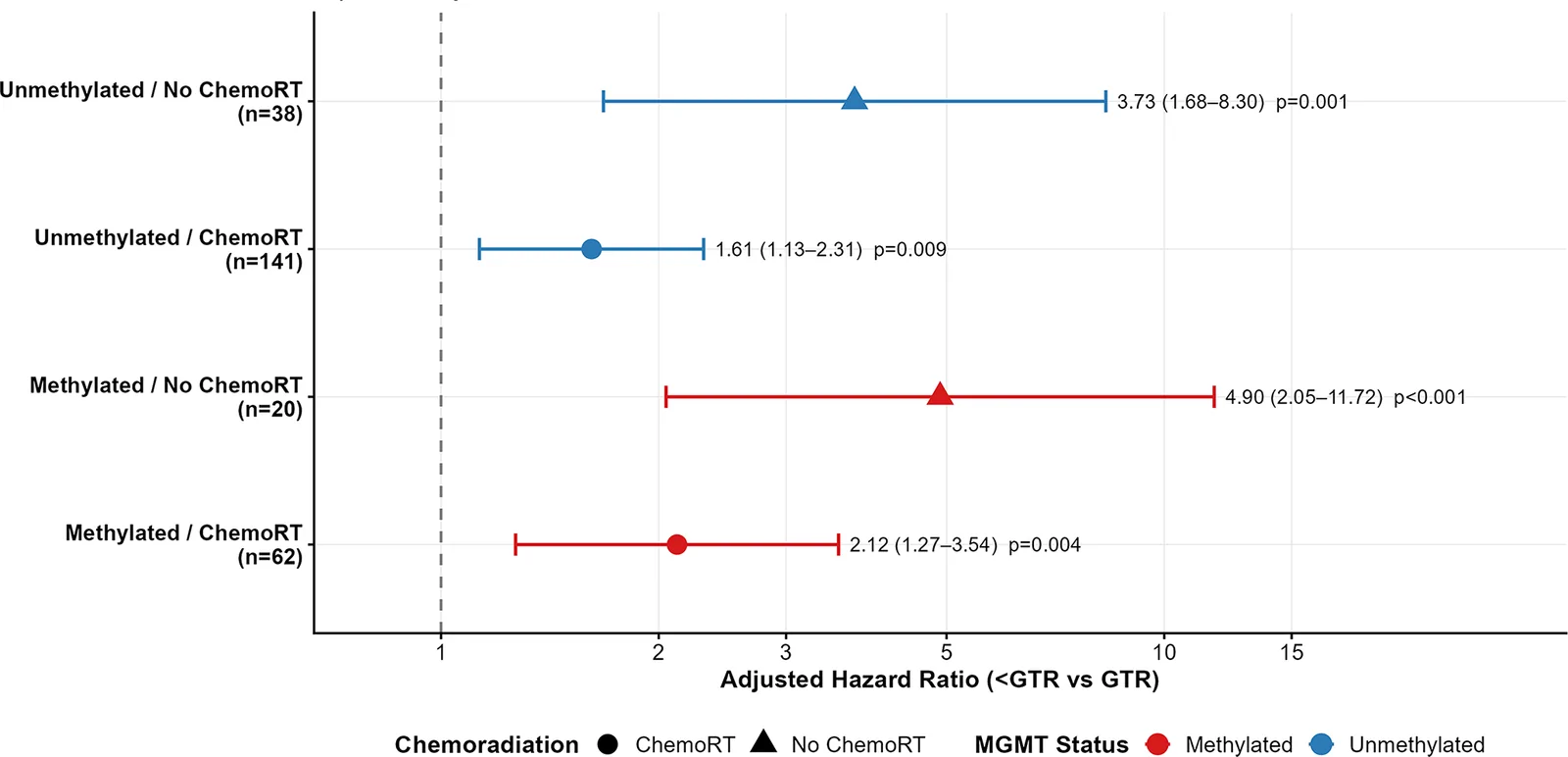
Forest plot of model-derived adjusted hazard ratios for < GTR versus GTR within each MGMT × chemoradiation stratum. Estimates were derived from the reduced two-way interaction Cox model adjusted for age per 10-year increase, KPS ≥ 90, deep/corpus callosum location, and mFI-5 ≥ 2. Horizontal bars represent 95% confidence intervals, and the dashed vertical line indicates HR = 1. Abbreviations: *ChemoRT*, chemoradiation; *CI*, confidence interval; *GTR*, gross total resection; *HR*, hazard ratio; *KPS*, Karnofsky Performance Status; *mFI-5*, 5-factor modified frailty index; *MGMT*, O^6^-methylguanine-DNA methyltransferase; *< GTR*, less-than-gross-total resection

Model-derived stratum-specific contrasts were calculated from the reduced two-way interaction Cox model and revealed that < GTR remained associated with worse survival than GTR in all four MGMT × ChemoRT strata. The adjusted HR for < GTR versus GTR was 3.73 in the unmethylated/no-ChemoRT stratum (95% CI 1.68–8.30, *p* = 0.001), 1.61 in the unmethylated/ChemoRT stratum (95% CI 1.13–2.31, *p* = 0.009), 4.90 in the methylated/no-ChemoRT stratum (95% CI 2.05–11.72, *p* < 0.001), and 2.12 in the methylated/ChemoRT stratum (95% CI 1.27–3.54, *p* = 0.004). Model-derived adjusted HRs are shown in Fig. 3, and the corresponding interaction visualization in Supplementary Fig. 2, which shows that the attenuation of the < GTR versus GTR association among ChemoRT-treated patients was similar across MGMT promoter status.

The full Cox model including the EOR × MGMT × ChemoRT three-way interaction is provided in Supplementary Table 3, and univariable Cox models for individual predictors are provided in Supplementary Table 4.

Sensitivity analyses retaining only patients who survived more than 6 weeks after diagnosis showed consistently elevated HRs for < GTR versus GTR across all MGMT × ChemoRT strata. In this cohort, adjusted HRs for < GTR versus GTR remained elevated in the unmethylated/no-ChemoRT stratum (HR 2.90, 95% CI 1.19–7.08, *p* = 0.019), unmethylated/ChemoRT stratum (HR 1.57, 95% CI 1.09–2.26, *p* = 0.016), methylated/no-ChemoRT stratum (HR 4.01, 95% CI 1.56–10.33, *p* = 0.004), and methylated/ChemoRT stratum (HR 2.17, 95% CI 1.29–3.65, *p* = 0.004). However, the EOR × ChemoRT interaction was no longer statistically significant (*p* = 0.150). Sensitivity results are shown in Supplementary Table 5. Another sensitivity analysis with more granular EOR variables included 101 GTR, 87 NTR/STR, and 73 MR/biopsy patients. NTR and STR did not significantly differ in survival (median OS, 11.4 vs. 5.1 months; HR 1.23, 95% CI 0.73–2.07; *p* = 0.443), and MR and biopsy did not differ significantly either (median OS, 1.7 vs. 3.0 months; HR 0.98, 95% CI 0.45–2.15; *p* = 0.958), and they were therefore pooled. Overall survival demonstrated a pattern across GTR, NTR/STR, and MR/biopsy (median OS, 15.6, 10.6, and 2.8 months, respectively; log-rank *p* < 0.001). In the adjusted model, hazard ratios versus GTR were 1.63 for NTR/STR (95% CI 1.20–2.21; *p* = 0.002) and 6.12 for MR/biopsy (95% CI 3.89–9.64; *p* < 0.001). After excluding MR/biopsy, NTR/STR was associated with worse survival than GTR (HR 1.67, 95% CI 1.22–2.28; *p* = 0.001). Three-group Kaplan-Meier survival was significantly different within each MGMT × ChemoRT stratum. In an adjusted analysis, MR/biopsy was associated with worse survival than GTR in all four strata. NTR/STR was significantly associated with worse survival in three strata and was only directionally worse in the unmethylated/ChemoRT stratum (HR 1.30, 95% CI 0.87–1.95; *p* = 0.199). The results of this analysis are demonstrated in Supplementary Figs. 3, 4 and Supplementary Tables 6, 7.

Proportional hazards diagnostics for the reduced two-way Cox model did not identify violations for any individual term, and the global test was non-significant (*p* = 0.181).

## Discussion

In this retrospective, single-center cohort of 261 patients with newly diagnosed IDH-wildtype glioblastoma, GTR was associated with longer overall survival than < GTR across all MGMT promoter methylation and chemoradiation strata. The key finding was that among MGMT-methylated patients receiving chemoradiation, median OS was 17.02 months after GTR versus 10.65 months after < GTR, and the model-derived adjusted HR for < GTR versus GTR was 2.12. Importantly, these estimates should be interpreted as associations, and not as causal effects, as the no-ChemoRT strata were small. Granular EOR sensitivity analyses demonstrated progressively worse survival from GTR to NTR/STR to MR/biopsy overall, with NTR/STR remaining associated with worse survival after MR/biopsy cases were excluded. In addition, the relative EOR-survival association appeared smaller among patients receiving chemoradiation, but this modification was exploratory and did not remain significant in the 6-week sensitivity analysis.

Our findings fit within prior literature which demonstrated that EOR, molecular variables, and adjuvant therapy all contribute to survival. Indeed, combination temozolomide with radiation improves survival, and MGMT promoter methylation predicts greater benefit [5, 6, 22]. However, in the present study, the MGMT-associated survival benefit was modest, but this should not be interpreted as evidence that MGMT is unimportant. As such, this study intended to evaluate the EOR-survival association across MGMT and chemoradiation strata, rather than isolate the independent impact of MGMT on chemotherapeutic benefit. Numerous surgical series, volumetric studies, and 5-ALA trials have linked postoperative tumor burden with survival and progression, although many studies came before routine molecular classification and may have heterogeneity in EOR definitions [8–11, 23]. Our study provides an updated, molecularly aware institutional analysis focused on whether the GTR-survival association remains detectable after stratification by both MGMT status and receipt of chemoradiation.

A prior National Cancer Database (NCDB) study of 27,858 IDH-wildtype glioblastoma patients found a GTR-associated survival advantage overall and in several subgroups, but not among MGMT-methylated patients receiving chemoradiation [19]. However, our methylated/ChemoRT subgroup retained a significant model-derived adjusted survival association that favored GTR. The studies may differ due to registry EOR coding, treatment coding, and covariate granularity. Our EOR classification was confirmed using early postoperative MRI and operative records, and the treatment classification was based on chart-confirmed initiation. Additionally, our model included preoperative KPS, mFI-5, and an imaging-defined tumor location variable. By contrast, the NCDB has a much greater sample size and generalizability but relies on potentially heterogeneous registry-based surgical and treatment fields and does not abstract the same imaging and functional variables. These differences may help explain, but not entirely establish, why the studies differed, and our findings should therefore be viewed as a complementary analysis of Parker et al. Our study supports the hypothesis that residual enhancing tumor may retain prognostic relevance in all subgroups of IDH-wildtype glioblastoma. Other studies similarly caution against assuming that MGMT methylation or chemoradiation offsets residual tumor. Mareike et al. reported that MGMT methylation improved outcomes among IDH-wildtype glioblastoma patients with postoperative residual tumor but did not fully compensate for the survival disadvantage associated with subtotal resection [17].

Prior EOR studies are broadly consistent with this interpretation. Gessler et al. found that GTR and MGMT promoter methylation were independent prognosticators in newly diagnosed IDH-wildtype glioblastoma, Marchi et al. found that MGMT methylation was particularly prognostic when residual tumor remained, and Incekara et al. found that maximal resection and minimal residual enhancing tumor volume were associated with longer OS in IDH-wildtype GBM [24–26]. Modern analyses emphasize residual tumor burden, which includes contrast-enhancing and non-contrast-enhancing tumor resection, Response Assessment in Neuro-Oncology (RANO) resect classification, and residual tumor volume as potentially more prognostically informative than EOR percentage alone [4, 14, 15, 27, 28]. Other RANO data also suggest that complete contrast-enhancing resection may differ prognostically even from near-total contrast-enhancing resection with ≤ 1 cm³ residual enhancement [29]. Together, these studies support the premise that residual enhancing tumor burden may remain prognostically relevant in favorable MGMT/ChemoRT contexts.

The interaction analysis should not be overinterpreted. The full model did not support a three-way EOR × MGMT × ChemoRT interaction, and the reduced model did not provide statistical evidence that the EOR-survival association differed by MGMT status alone. The nonsignificant MGMT × ChemoRT interaction should also be interpreted cautiously, as this term was tested within an EOR-based model adjusted for EOR, age, KPS, location, and frailty, rather than as a standalone test of MGMT biology on temozolomide response. The individual EOR × ChemoRT term suggested a smaller relative EOR association among patients receiving chemoradiation, but the full two-way interaction block was not significant and the EOR × ChemoRT signal was not significant in the sensitivity analysis. Because ChemoRT was a post-surgical variable, this interaction may reflect survivor-treatment selection or residual confounding. Nonetheless, these data are consistent with maximal safe resection when technically feasible [16].

Baseline imbalance remains central to interpretation. Patients undergoing GTR were younger, had higher KPS, were more likely to receive chemoradiation, and had no deep/corpus callosum tumors. This reflects surgical selection, as deep, callosal, or eloquent tumors are less safely resectable and are less likely to undergo GTR [30]. Separately, periventricular or subventricular-zone contact has been associated with worse outcomes [31–33]. Because no GTR patients had deep/corpus callosum tumors, the adjusted model lacks support for estimating the association of GTR within that location stratum; therefore, residual confounding is likely.

After excluding deaths within 6 weeks, < GTR remained associated with worse survival in all four MGMT × ChemoRT strata, but the EOR × ChemoRT interaction was no longer significant. This analysis suggests that the EOR association was not solely driven by deaths occurring during the first 6 weeks. Importantly, it does not fully resolve treatment selection or immortal-time bias because inclusion was based on survival after the diagnosis. Residual time-related biases therefore likely remain [34]. The granular EOR sensitivity analysis addresses heterogeneity within < GTR. Survival was shorter across GTR, NTR/STR, and MR/biopsy overall, and the association between NTR/STR and worse survival persisted after MR/biopsy cases were removed. However, NTR/STR versus GTR was not statistically significant in the unmethylated/ChemoRT stratum, and comparisons often included wide confidence intervals, demonstrating heterogeneity within incomplete resection categories.

Strengths include restriction to IDH-wildtype glioblastoma, integration of EOR with MGMT and chemoradiation status, model-based stratum-specific contrasts, direct evaluation of a clinically relevant uncertainty, and robust longitudinal follow-up within a single-institution practice setting. Limitations include the retrospective single-institution design, baseline imbalance between GTR and < GTR groups, and lack of common support for deep/corpus callosum tumors. Additionally, the subgroup sizes substantially limit key conclusions, as the no-ChemoRT groups included only 5 GTR patients in each group, leading to wide confidence intervals and limited power for meaningful interaction tests. As such, significant associations should not be overinterpreted. Furthermore, generalizability remains a concern due to the nature of the single-center design. Although the granular EOR sensitivity analysis supported the main finding, subgroups became underpowered as they were further stratified. Chemoradiation was defined by treatment received after diagnosis, so additional confounding by indication and immortal-time bias remain even after the 6-week sensitivity analysis. Future studies should incorporate standardized preoperative contrast-enhancing as well as non-contrast-enhancing tumor volumes and their postoperative residuals. MGMT was analyzed as a binary clinical variable despite evidence that quantitative methylation values may influence prognosis [35].

## Conclusion

In this single-institution cohort of newly diagnosed IDH-wildtype glioblastoma, GTR was associated with longer overall survival than < GTR across all MGMT promoter methylation and chemoradiation strata. Granular EOR analyses showed an overall pattern from GTR to NTR/STR to MR/biopsy, although not every NTR/STR contrast was statistically significant. Our findings are consistent with considering maximal safe resection when clinically feasible across all MGMT and chemoradiation subgroups, but they should be interpreted cautiously because of small subgroups, patient selection, residual confounding, and immortal-time bias. These results should be validated in larger, imaging-rich molecular cohorts.

## Supplementary Information

Below is the link to the electronic supplementary material.


Supplementary Material 1


## Data Availability

De-identified data may be available from the corresponding author upon reasonable request.
