# Supplementary material for: Extent of resection and survival in IDH-wildtype glioblastoma: interaction with MGMT status and chemoradiation
Source: J Neurooncol. 2026 Jul 23;179(1):10. doi: 10.1007/s11060-026-05725-x (PMC13395848; doi:10.1007/s11060-026-05725-x)
Supplement: Supplementary file 1 — Supplementary Material 1 [file 11060_2026_5725_MOESM1_ESM.docx]

**Online Resource 1**

Extent of resection and survival in IDH-wildtype glioblastoma: interaction with MGMT status and chemoradiation

Journal: Journal of Neuro-Oncology

Noah B. Drewes, BS^1†^, Lara Koutah, MS^2†^, Rishi Jain, BS^2^, Jack Carnduff, BS^1^, Kayla Chin, MD^1^, Kristin R. Delfino, PhD^3^, Jeffrey W. Cozzens, MD^1^, Bruce M. Frankel, MD^1^

^1^Department of Surgery, Division of Neurosurgery, Southern Illinois University School of Medicine, Springfield, IL, USA

^2^Department of Neurological Surgery, Feinberg School of Medicine, Northwestern University, Chicago, IL 60611, USA

^3^Center for Clinical Research, Southern Illinois University School of Medicine, Springfield, IL, USA

^†^ Noah B. Drewes and Lara Koutah contributed equally as first authors.

**Corresponding Author:**

Noah B. Drewes, BS

Department of Surgery, Division of Neurosurgery

Southern Illinois University School of Medicine

801 N. Rutledge St., PO Box 19620, Springfield, IL 62702, United States

Telephone: +1 217-303-1561

Email: ndrewes45@siumed.edu

ORCID iD: 0009-0006-2636-0516

**Contents**

Supplementary Figure 1. Overall Kaplan-Meier survival by extent of resection

Supplementary Figure 2. Adjusted EOR interaction visualization by MGMT status and chemoradiation

Supplementary Figure 3: Kaplan-Meier curves using GTR, NTR/STR, and MR/biopsy within each MGMT × ChemoRT stratum.

Supplementary Figure 4: Model-derived hazard ratios for NTR/STR and MR/biopsy versus GTR within each stratum.

Supplementary Table 1. Baseline characteristics by MGMT x chemoradiation stratum

Supplementary Table 2. Unadjusted Kaplan-Meier median OS by MGMT x chemoradiation stratum and EOR

Supplementary Table 3. Full three-way Cox model

Supplementary Table 4. Univariable Cox models

Supplementary Table 5. Sensitivity analysis: patients surviving >6 weeks

Supplementary Table 6: Sensitivity analysis: granular EOR analyses

Supplementary Table 7: Stratum-specific median OS and adjusted granular EOR hazard ratios

**Supplementary Figure 1**


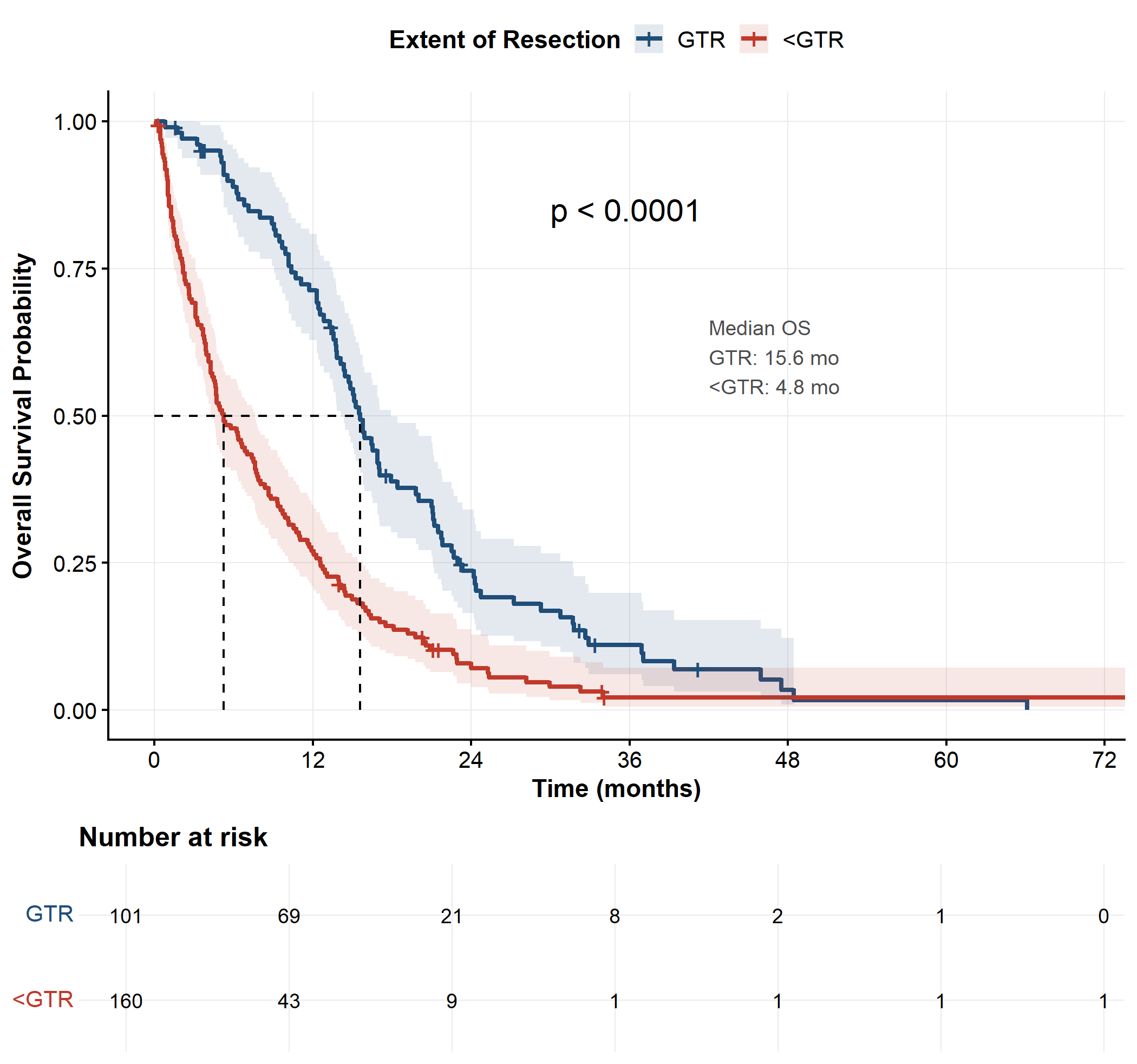


Overall Kaplan-Meier survival by extent of resection in the full cohort. The log-rank p value compares GTR versus <GTR. Shaded bands represent 95% confidence intervals and the number-at-risk table is shown below the curves. Abbreviations: *EOR*, extent of resection; *GTR*, gross total resection; *OS*, overall survival; *<GTR*, less-than-gross-total resection.

**Supplementary Figure 2**


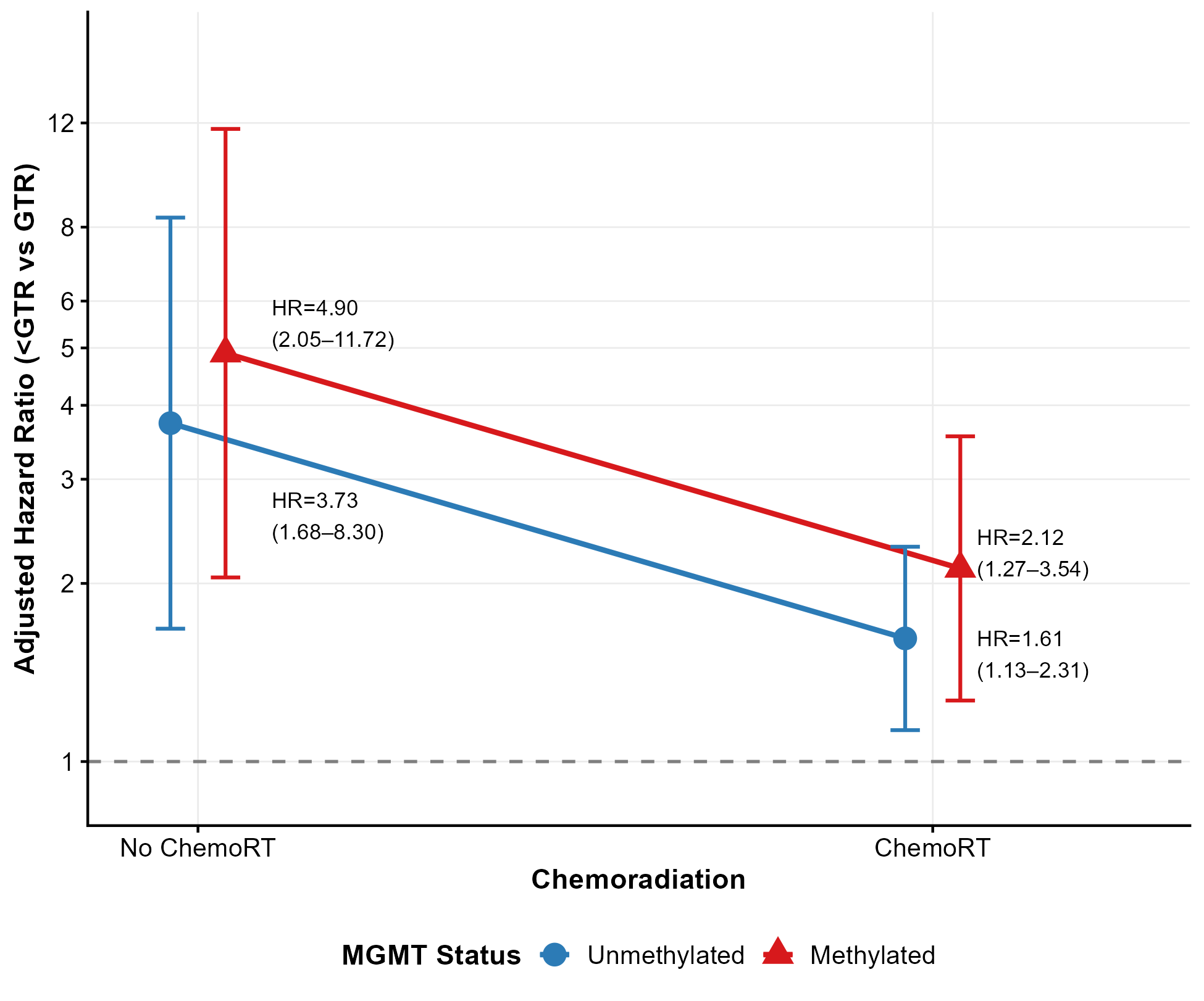


Interaction visualization showing model-derived adjusted hazard ratios for <GTR versus GTR by chemoradiation status, with separate lines for MGMT promoter methylation status. Estimates are derived from the reduced two-way interaction Cox model. Abbreviations: *ChemoRT,* chemoradiation; *EOR*, extent of resection; *GTR*, gross total resection; *MGMT*, O6-methylguanine-DNA methyltransferase; *<GTR*, less-than-gross-total resection.

**Supplementary Figure 3**


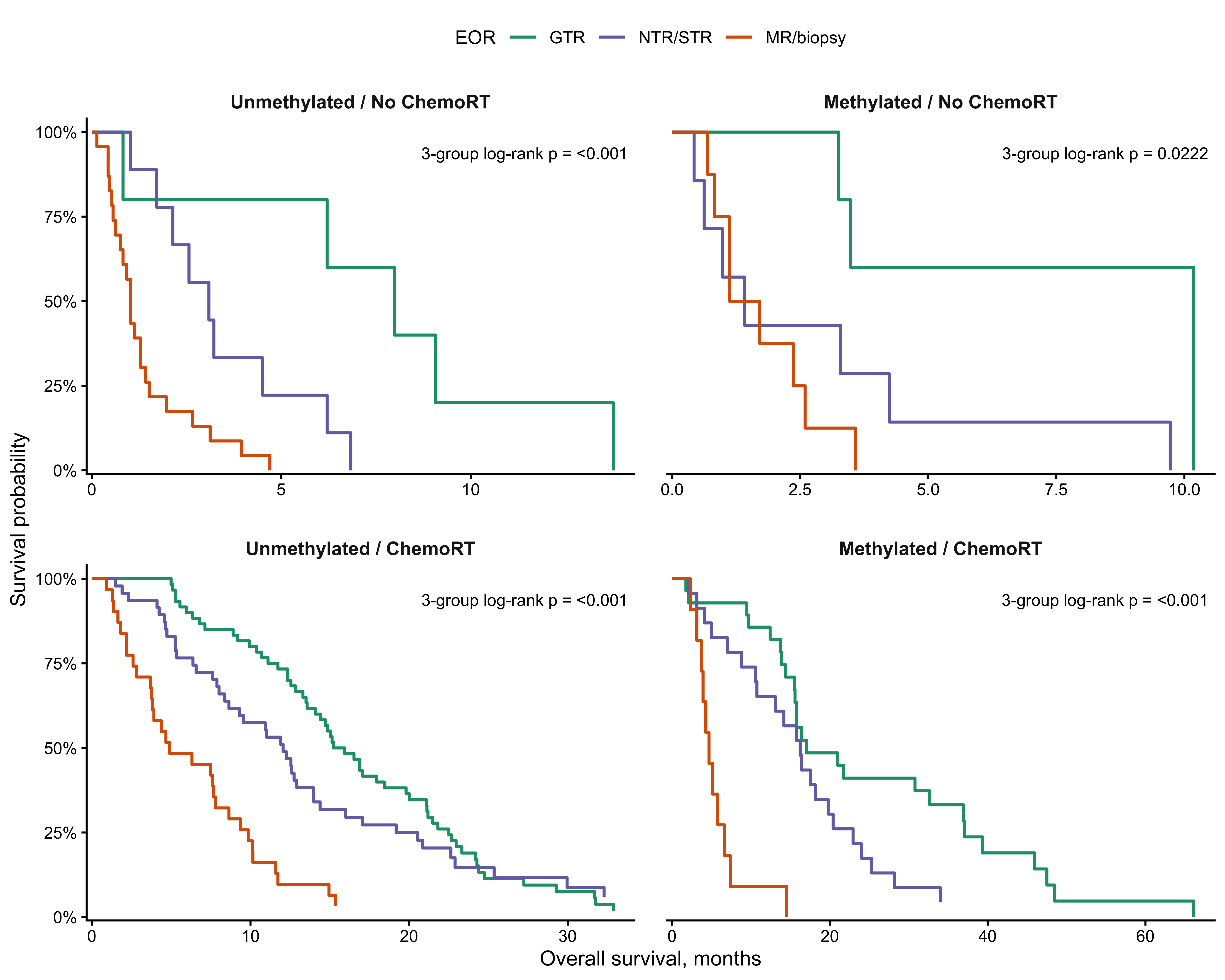


Kaplan-Meier overall survival curves using three EOR categories within each MGMT and chemoradiation stratum. P values are from three-group log-rank tests. Abbreviations: *ChemoRT*, chemoradiation; *EOR*, extent of resection; *GTR*, gross total resection; *MGMT*, O6-methylguanine-DNA methyltransferase; *MR*, minimal resection; *NTR*, near-total resection; *OS*, overall survival; *STR*, subtotal resection.

**Supplementary Figure 4**


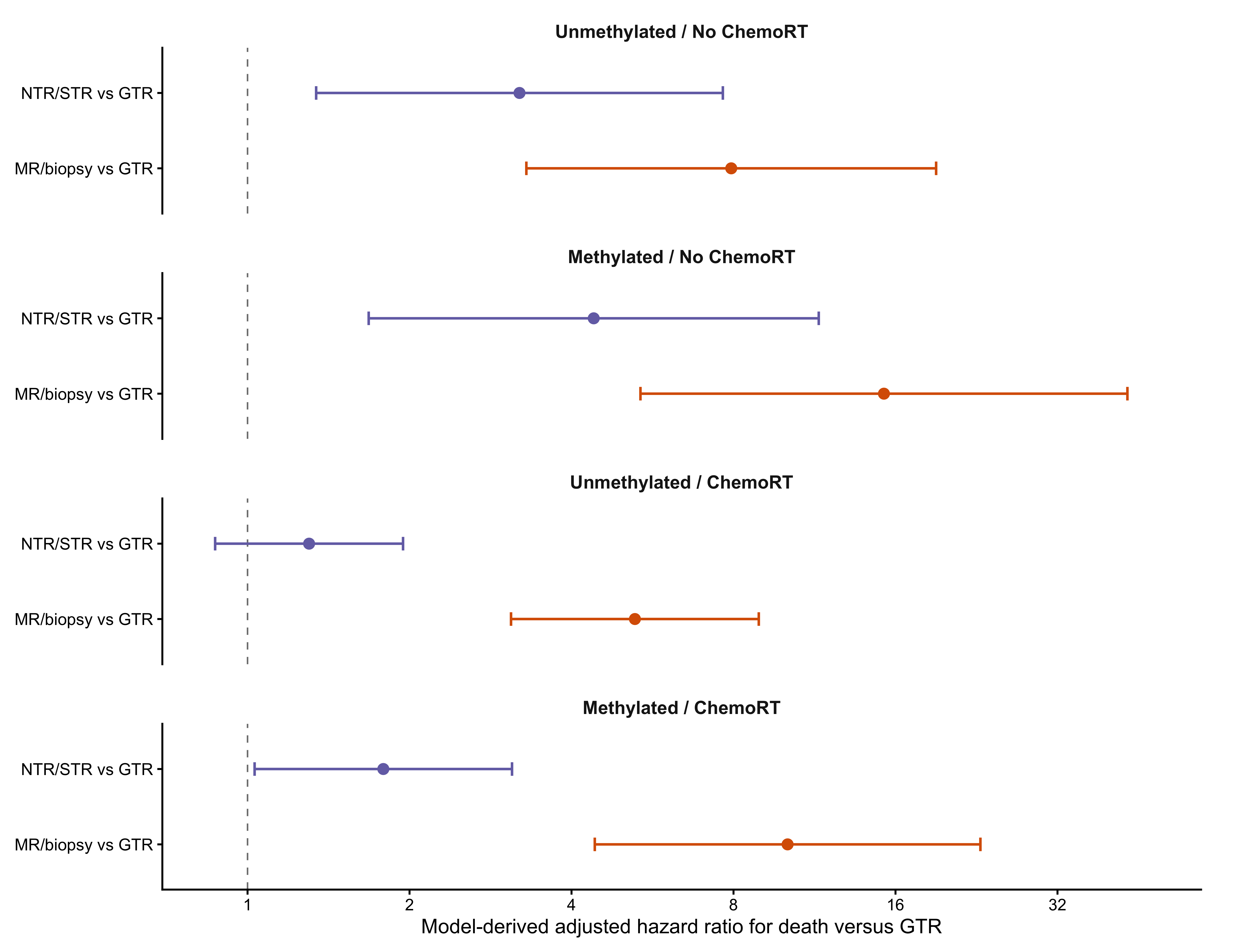


Forest plot of model-derived adjusted hazard ratios for NTR/STR and MR/biopsy versus GTR within each MGMT and chemoradiation stratum. Horizontal bars represent 95% confidence intervals, and the dashed vertical line indicates HR = 1. Abbreviations: *ChemoRT*, chemoradiation; *EOR*, extent of resection; *GTR*, gross total resection; *HR*, hazard ratio; *MGMT*, O6-methylguanine-DNA methyltransferase; *MR*, minimal resection; *NTR*, near-total resection; *STR*, subtotal resection.

**Supplementary Table 1. Baseline Characteristics by MGMT × Chemoradiation Stratum**

| **Characteristic** | **UM / No ChemoRT (n=38)** | **UM / ChemoRT (n=141)** | **M / No ChemoRT (n=20)** | **M / ChemoRT (n=62)** | **P-Value** |
| --- | --- | --- | --- | --- | --- |
| Age, years, median [IQR] | 75.2 [67.3-80.9] | 64.7 [57.1-70.9] | 74.5 [72.1-85.1] | 66.0 [59.3-71.1] | <0.001^*^ |
| KPS ≥90, n (%) | 24 (63.2) | 123 (87.2) | 12 (60.0) | 55 (88.7) | 0.001^*^ |
| mFI-5 ≥2, n (%) | 13 (34.2) | 26 (18.4) | 11 (55.0) | 18 (29.0) | 0.002^*^ |
| Deep or CC location, n (%) | 14 (36.8) | 18 (12.8) | 3 (15.0) | 7 (11.3) | 0.003^*^ |
| GTR, n (%) | 5 (13.2) | 63 (44.7) | 5 (25.0) | 28 (45.2) | 0.002^*^ |

Abbreviations: *CC*, corpus callosum; *ChemoRT*, chemoradiation; *GTR*, gross total resection; *IQR*, interquartile range; *KPS*, Karnofsky Performance Status; *M*, methylated; *mFI-5*, 5-factor modified frailty index; *MGMT*, O6-methylguanine-DNA methyltransferase; *UM*, unmethylated.

* denotes p < 0.05.

**Supplementary Table 2. Unadjusted Kaplan-Meier Median OS by MGMT × ChemoRT Stratum and EOR**

| **Stratum** | **EOR** | **N** | **Events** | **Median OS, months** | **95% CI lower** | **95% CI upper** | **Log-rank P-Value** |
| --- | --- | --- | --- | --- | --- | --- | --- |
| UM / No ChemoRT | GTR | 5 | 5 | 7.98 | 0.82 | NR | 0.002^*^ |
|  | <GTR | 33 | 32 | 1.28 | 0.92 | 2.14 |  |
| UM / ChemoRT | GTR | 63 | 57 | 15.59 | 13.5 | 19.81 | 0.001^*^ |
|  | <GTR | 78 | 72 | 8.64 | 6.57 | 11.01 |  |
| M / No ChemoRT | GTR | 5 | 4 | 10.18 | 3.25 | NR | 0.009^*^ |
|  | <GTR | 15 | 15 | 1.41 | 0.69 | 2.6 |  |
| M / ChemoRT | GTR | 28 | 25 | 17.02 | 15.51 | 36.93 | 0.001^*^ |
|  | <GTR | 34 | 33 | 10.65 | 5.12 | 16.23 |  |

NR = not reached within observed follow-up. Log-rank p-values shown per stratum.

Abbreviations: *ChemoRT*, chemoradiation; *CI*, confidence interval; *EOR*, extent of resection; *GTR*, gross total resection; *KM*, Kaplan-Meier; *M*, methylated; *MGMT*, O6-methylguanine-DNA methyltransferase; *NR*, not reached; *OS*, overall survival; *UM*, unmethylated; *<GTR*, less-than-gross-total resection.

* denotes p < 0.05.

**Supplementary Table 3. Full Three-Way Cox Model**

| **Parameter** | **β** | **SE** | **HR** | **95% CI lower** | **95% CI upper** | **P-Value** |
| --- | --- | --- | --- | --- | --- | --- |
| EOR: <GTR vs GTR | 1.576 | 0.507 | 4.84 | 1.79 | 13.07 | 0.002^*^ |
| MGMT methylated | -0.014 | 0.678 | 0.99 | 0.26 | 3.74 | 0.983 |
| Chemoradiation | -1.027 | 0.484 | 0.36 | 0.14 | 0.93 | 0.034^*^ |
| EOR × MGMT | -0.432 | 0.744 | 0.65 | 0.15 | 2.79 | 0.561 |
| EOR × Chemoradiation | -1.136 | 0.533 | 0.32 | 0.11 | 0.92 | 0.033^*^ |
| MGMT × Chemoradiation | -0.617 | 0.724 | 0.54 | 0.13 | 2.22 | 0.395 |
| EOR × MGMT × Chemoradiation | 0.838 | 0.815 | 2.31 | 0.47 | 11.4 | 0.304 |
| Age, per 10-year increment | 0.183 | 0.063 | 1.2 | 1.06 | 1.36 | 0.004^*^ |
| KPS ≥90 vs <90 | -0.508 | 0.188 | 0.6 | 0.42 | 0.87 | 0.007^*^ |
| Deep or CC location | 0.697 | 0.203 | 2.01 | 1.35 | 2.99 | 0.001^*^ |
| mFI-5 ≥2 vs <2 | 0.119 | 0.164 | 1.13 | 0.82 | 1.55 | 0.467 |

Abbreviations: *β*, regression coefficient; *CC*, corpus callosum; *CI*, confidence interval; *EOR*, extent of resection; *GTR*, gross total resection; *HR*, hazard ratio; *KPS*, Karnofsky Performance Status; *mFI-5*, 5-factor modified frailty index; *MGMT*, O6-methylguanine-DNA methyltransferase; *SE*, standard error; *<GTR*, less-than-gross-total resection.

* denotes p < 0.05.

**Supplementary Table 4. Univariable Cox Models**

| **Variable** | **HR** | **95% CI lower** | **95% CI upper** | **P-Value** |
| --- | --- | --- | --- | --- |
| EOR: <GTR vs GTR | 2.24 | 1.72 | 2.92 | <0.001^*^ |
| MGMT methylated vs unmethylated | 0.76 | 0.58 | 1 | 0.05 |
| Chemoradiation: yes vs no | 0.13 | 0.09 | 0.18 | <0.001^*^ |
| Age, per 10-year increment | 1.39 | 1.23 | 1.56 | <0.001^*^ |
| KPS ≥90 vs <90 | 0.41 | 0.29 | 0.57 | <0.001^*^ |
| Deep or CC location vs other | 3.73 | 2.61 | 5.33 | <0.001^*^ |
| mFI-5 ≥2 vs <2 | 1.6 | 1.2 | 2.14 | 0.002^*^ |

Abbreviations: *CC*, corpus callosum; *CI*, confidence interval; *EOR*, extent of resection; *GTR*, gross total resection; *HR*, hazard ratio; *KPS*, Karnofsky Performance Status; *mFI-5*, 5-factor modified frailty index; *MGMT*, O6-methylguanine-DNA methyltransferase; *<GTR*, less-than-gross-total resection.

* denotes p < 0.05.

**Supplementary Table 5. Sensitivity Analysis: Patients Surviving >6 Weeks (n=232)**

| **Panel A: Interaction LRTs** | | | |
| --- | --- | --- | --- |
| **Model Comparison** | **LRT χ²** | **df** | **P-Value** |
| Full (3-way) vs reduced (2-way) | 1.62 | 1 | 0.204 |
| EOR × MGMT | 1.3 | 1 | 0.254 |
| EOR × Chemoradiation | 2.08 | 1 | 0.15 |
| MGMT × Chemoradiation | 0.18 | 1 | 0.67 |
| All two-way (block) | 3.3 | 3 | 0.348 |

| **Panel B: Stratum-Specific Adjusted HRs for <GTR vs GTR** | | | | | |
| --- | --- | --- | --- | --- | --- |
| **Stratum** | **N** | **Adjusted HR** | **95% CI lower** | **95% CI upper** | **P-Value** |
| Unmethylated / No chemoradiation | 19 | 2.9 | 1.19 | 7.08 | 0.019^*^ |
| Unmethylated / Chemoradiation | 138 | 1.57 | 1.09 | 2.26 | 0.016^*^ |
| Methylated / No chemoradiation | 13 | 4.01 | 1.56 | 10.33 | 0.004^*^ |
| Methylated / Chemoradiation | 62 | 2.17 | 1.29 | 3.65 | 0.004^*^ |

Abbreviations: *CI*, confidence interval; *EOR*, extent of resection; *GTR*, gross total resection; *HR*, hazard ratio; *LRT*, likelihood-ratio test; *M*, methylated; *MGMT*, O6-methylguanine-DNA methyltransferase; *UM*, unmethylated; *<GTR,* less-than-gross-total resection.

* denotes p < 0.05.

**Supplementary Table 6. Granular EOR Sensitivity Analyses**

| **Panel A. Exploratory comparisons within pooled categories** | | | | | |
| --- | --- | --- | --- | --- | --- |
| **Comparison** | **Median OS, group 1** | **Median OS, group 2** | **HR** | **95% CI** | **P-Value** |
| NTR vs STR | 11.4 months | 5.1 months | 1.23 | 0.73-2.07 | 0.443 |
| MR vs biopsy-only | 1.7 months | 3.0 months | 0.98 | 0.45-2.15 | 0.958 |

| **Panel B. Overall three-category EOR analysis** | | | | | | |
| --- | --- | --- | --- | --- | --- | --- |
| **EOR group** | **N** | **Events** | **Median OS, months** | **Adjusted HR** | **95% CI** | **P-Value** |
| GTR | 101 | 91 | 15.6 | Reference | - | - |
| NTR/STR | 87 | 80 | 10.6 | 1.63 | 1.20-2.21 | 0.002* |
| MR/biopsy | 73 | 72 | 2.8 | 6.12 | 3.89-9.64 | <0.001* |

| **Panel C. Analysis restricted to GTR versus NTR/STR** | | | | | |
| --- | --- | --- | --- | --- | --- |
| **Analysis** | **N** | **Events** | **Adjusted HR** | **95% CI** | **P-Value** |
| GTR and NTR/STR only | 188 | 171 | 1.67 | 1.22-2.28 | 0.001* |

Abbreviations: *ChemoRT*, chemoradiation; *CI*, confidence interval; *EOR*, extent of resection; *GTR*, gross total resection; *HR*, hazard ratio; *KPS*, Karnofsky Performance Status; *mFI-5*, 5-factor modified frailty index; *MGMT*, O6-methylguanine-DNA methyltransferase; *MR*, minimal resection; *NTR*, near-total resection; *OS*, overall survival; *STR*, subtotal resection.

* denotes p < 0.05.

**Supplementary Table 7. Granular EOR Analyses by MGMT × Chemoradiation Stratum**

| **Panel A. Kaplan-Meier median OS by three-category EOR group** | | | | |
| --- | --- | --- | --- | --- |
| **Stratum** | **GTR median OS** | **NTR/STR median OS** | **MR/biopsy median OS** | **3-group log-rank P-Value** |
| Unmethylated / No ChemoRT | 8.0 | 3.1 | 1.0 | <0.001* |
| Methylated / No ChemoRT | 10.2 | 1.4 | 1.4 | 0.022* |
| Unmethylated / ChemoRT | 15.6 | 12.1 | 4.9 | <0.001* |
| Methylated / ChemoRT | 17.0 | 16.2 | 4.7 | <0.001* |

| **Panel B. Model-derived adjusted contrasts versus GTR** | | | | |
| --- | --- | --- | --- | --- |
| **Stratum** | **EOR contrast** | **Adjusted HR** | **95% CI** | **P-Value** |
| Unmethylated / No ChemoRT | NTR/STR vs GTR | 3.20 | 1.34-7.64 | 0.009* |
|  | MR/biopsy vs GTR | 7.92 | 3.30-19.04 | <0.001* |
| Methylated / No ChemoRT | NTR/STR vs GTR | 4.40 | 1.68-11.52 | 0.003* |
|  | MR/biopsy vs GTR | 15.23 | 5.37-43.16 | <0.001* |
| Unmethylated / ChemoRT | NTR/STR vs GTR | 1.30 | 0.87-1.95 | 0.199 |
|  | MR/biopsy vs GTR | 5.25 | 3.09-8.91 | <0.001* |
| Methylated / ChemoRT | NTR/STR vs GTR | 1.79 | 1.03-3.10 | 0.039* |
|  | MR/biopsy vs GTR | 10.08 | 4.42-23.01 | <0.001* |

Abbreviations: *ChemoRT*, chemoradiation; *CI*, confidence interval; *EOR*, extent of resection; *GTR*, gross total resection; *HR*, hazard ratio; *KPS*, Karnofsky Performance Status; *mFI-5,* 5-factor modified frailty index; *MGMT*, O6-methylguanine-DNA methyltransferase; *MR*, minimal resection; *NTR*, near-total resection; *OS*, overall survival; *STR*, subtotal resection.

* denotes p < 0.05.
